# The Impact of Young Age for Prognosis by Subtype in Women with Early Breast Cancer

**DOI:** 10.1038/s41598-017-10414-x

**Published:** 2017-09-14

**Authors:** Weibin Lian, Fangmeng Fu, Yuxiang Lin, Minjun Lu, Boyang Chen, Peidong Yang, Bangwei Zeng, Meng Huang, Chuan Wang

**Affiliations:** 1 Department of General Surgery, Fujian Medical University Union Hospital, Fuzhou, Fujian Province 350001 China; 2 Department of Thoracic Surgery, Fujian Medical University Union Hospital, Fuzhou, Fujian Province 350001 China; 3 Fujian Center for Disease Control and Prevention, Fuzhou, Fujian Province 350001 China

## Abstract

Young age (≤40 years) use to be considered an independent risk factor for the prognosis of women with early-stage breast cancer. We conducted a retrospective analysis to investigate this claim in a population of young patients who were stratified by molecular subtype. We identified 2,125 women with stage I to III breast cancer from the Fujian Medical University Union Hospital. Multivariable Cox proportional hazards models were used to analyze the relationship between age groups stratified by molecular subtype and 5-year disease-free survival (DFS), 5-year distant metastasis-free survival (DMFS), and 5-year breast cancer-specific survival (BCSS). Median follow-up time was 77 months. Patients ≤40 years of age presented with a significantly worse 5-year DFS and 5-year DMFS. In stratified analyses, young women with luminal A subtype disease were associated with a worse 5-year DFS, 5-year DMFS, and 5-year BCSS. Women with luminal B (Her2−) tumors showed a decrease in 5-year DFS and 5-year DMFS. Our findings support the hypothesis that young age seems to be an independent risk factor for the prognosis for breast cancer patients with the luminal A and luminal B (Her2−) subtypes but not in those with luminal B (Her2+), Her2 over-expression, and triple-negative disease.

## Introduction

Breast cancer is the most common cause of death from carcinomas in women in developing countries, and it is the second most common cause in women in developed countries^1^. Breast cancer accounts for approximately 5–7% in developed countries among younger women (≤40 years old), however, it is accounts for about 20% in the same population in China^2–4^. This suggests that different geographic regions and ethnicities have different age structure.

Several large studies have reported that young age seems to indicate an unfavorable prognosis at the diagnosis of breast cancer, and performs as an independent risk factor in patients for a higher risk of recurrence and death^5–9^. Previous studies have suggested that younger women develop cancers with more aggressive biological features and are more commonly diagnosed with larger tumors, lymphatic metastasis, higher grade disease, estrogen receptor-negative tumors, and over-expression of the human epidermal growth factor receptor 2 (HER2)^5, 10^.

Numerous studies have confirmed that there are distinct molecular subtypes of breast cancer, which are inextricably bound up with therapeutic response and prognosis^11–13^. At present, breast cancer is separated into at least four subtypes; luminal A, luminal B, basal-like, and HER2 over-expressing^14^. Desmedt *et al*. found that receptor phenotype, histologic grade and tumor cell proliferation can be substituted for the main molecular subtypes^15^. Younger women with breast cancer are more likely to develop more aggressive subtypes, which include a higher proportion of basal-like and HER2 over-expressing tumors that are associated with a poor prognosis^9, 16^. Nevertheless, recent studies have reported that in women with HER2 over-expression or triple-negative breast cancer, the risk of recurrence seems to be similar in younger women compared with older women when controlling for other conventional prognostic factors^17–19^. Based on the conflicting reports it seems that young age may not always be an independent risk factor for some molecular subtypes. Hence, it was incumbent upon us to determine the subtypes associated with a poorer prognosis for younger Chinese women.

This study retrospectively investigated the effect of age on the prognosis of breast cancer, and most importantly, explored whether young age is always an independent risk factor for DFS, DMFS, and BCSS in patients with different molecular subtypes of early-stage breast cancer. New diagnoses and treatment strategies for clinical use may become available after applying the results of this study.

## Results

### Patient characteristics and treatment

A total of 2,125 women with breast cancer were eligible for this study. Those younger than 40 years of age at the time of diagnosis accounted for twenty-five percent and the median follow-up time was 75 months. The characteristics and treatment of patients are summarized in Table 1. Younger patients showed a significant association with known worse prognostic variables such as lymph node metastasis, tumor stage III, and high Ki-67 expression. Furthermore, the most prevalent molecular subtypes in younger patients were luminal B (Her2+) and luminal B (Her2−), which were found to differ significantly from the reference group (p < 0.0001).

**Table 1:** Characteristics of 2125 patients by age group.

Age at diagnosis (years)					
	≤40	41–50	51–60	>60	P*
	N(%)	N(%)	N(%)	N(%)	
**Tumor sizes**					0.7257
≤2 cm	218 (40.2)	320 (42.2)	219 (39.5)	114 (42.4)	
>2 cm	324 (59.8)	439 (57.8)	336 (60.5)	155 (57.6)	
**Node statue**					0.0134
N0	265 (48.9)	423 (55.7)	287 (51.7)	160 (59.5)	
N+	277 (51.0)	336 (44.3)	268 (48.3)	109 (40.5)	
**Stage**					0.0126
I+II	394 (72.7)	590 (77.7)	418 (75.3)	222 (82.5)	
III	148 (27.3)	169 (22.3)	137 (24.7)	47 (17.5)	
**Histologic Grade**					0.3285
Low/moderate	378 (69.7)	529 (69.7)	397 (71.5)	192 (71.4)	
High	146 (27.0)	171 (22.5)	119 (21.4)	65 (24.2)	
unknown	18 (3.3)	59 (7.8)	39 (7.0)	12 (4.4)	
**ER status**					0.0026
positive	384 (70.9)	526 (69.3)	339 (61.1)	180 (66.9)	
negative	158 (29.1)	233 (30.7)	216 (38.9)	89 (33.1)	
**PR status**					<0.0001
positive	377 (69.6)	489 (64.4)	282 (50.8)	157 (58.4)	
negative	165 (30.4)	270 (35.6)	273 (49.2)	112 (41.6)	
**HER2 status**					0.0015
positive	162 (29.9)	229 (30.2)	199 (35.9)	61 (22.7)	
negative	380 (70.1)	530 (69.8)	356 (64.1)	208 (77.3)	
**Ki67**					0.0065
Low	168 (31.0)	283 (37.3)	201 (36.2)	124 (46.1)	
High	231 (42.6)	280 (36.9)	225 (40.5)	98 (36.4)	
unknown	143 (26.4)	196 (25.8)	129 (23.3)	47 (17.5)	
**Molecular subtype**					<0.0001
Luminal A	112 (20.7)	183 (24.1)	100 (18.0)	71 (26.4)	
Luminal B Her-2 (+)	92 (17.0)	114 (15.0)	72 (13.0)	24 (8.9)	
Luminal B Her-2 (−)	186 (34.3)	232 (30.6)	168 (30.3)	86 (32.0)	
HER-2 over- expression	70 (12.9)	114 (15.0)	127 (22.9)	36 (13.4)	
Triple negative	82 (15.1)	116 (15.3)	88 (15.8)	52 (19.3)	
**Chemotherapy**					<0.0001
Yes	526 (97.1)	741 (97.6)	539 (97.1)	182 (67.7)	
No	16 (2.9)	18 (2.4)	16 (2.9)	87 (32.3)	
**Endocrine-therapy**					<0.0001
Yes	354 (65.3)	477 (62.9)	294 (53.0)	143 (53.2)	
No	188 (34.7)	282 (37.1)	261 (47.0)	126 (46.8)	
**Trastuzumab**					0.2328
Yes	16 (2.9)	18 (2.4)	11 (2.0)	2 (0.7)	
No	526 (97.1)	741 (97.6)	544 (98.0)	267 (99.3)	

Abbreviations: ER, estrogen receptor; HER2, human epidermal growth factor receptor 2; PR, progesterone receptor.

*P value from χ^2^ test.

### Survival analysis (5-year DFS, 5-year DMFS, and 5-year BCSS) between age-groups

For the 2,125 breast cancer patients with available follow-up information who were analyzed, the median follow-up was 77 months. Younger women showed an inferior 5-year DFS, 5-year DMFS, and 5-year BCSS compared to the 41 to 50 year age group. With multivariable analysis using the Cox’s proportional hazards model after controlling for tumor stage, molecular subtype and treatment (chemotherapy, endocrine therapy, or trastuzumab), we found that the 5-year DFS of the younger group is 1.37-fold higher than for women from 41 to 50 year old at diagnosis (HR = 1.37, 95%CI 1.12–1.67, Table 2a). Younger women also have a worse 5-year DMFS (Table 2b). The 5-year breast cancer-special survival-rate was 84.9% in younger women, which is a worse outcome in contrast to the 41 to 50, 51 to 60, and >60 year old populations, 89.5%, 86.3%, and 85.9% respectively. However, the difference was only associated with a borderline increased risk (HR = 1.37, 95%CI 1.00–1.89, Table 2c). No significant difference was seen between women age 51 to 60 years (HR = 1.06, 95%CI 0.85–1.31) and those >60 years old (HR = 1.20, 95%CI 0.92–1.58) for 5-year DFS, 5-year DMFS, and 5-year BCSS compared with the 41 to 50 year old age group.

**Table 2:** Hazard Ratios and 95% CIs for 5-years DFS, 5-years DMFS and 5-years BCSS by age group.

**a. Hazard Ratios and 95% CIs for 5-years DFS by age group**				
Age group (years)	patient N (%)	5-years DFS N (%)	HR (95% CI)	HR (95% CI)*
≤40	542	339 (62.5)	1.45 (1.20 to 1.76)	1.37 (1.12 to 1.67)
41–50	759	551 (72.6)	1.00 (REF)	1.00 (REF)
51–60	555	381 (68.6)	1.18 (0.96 to 1.44)	1.06 (0.85 to 1.31)
>60	269	177 (65.8)	1.37 (0.99 to 1.62)	1.20 (0.92 to 1.58)
**b. Hazard Ratios and 95% CIs for 5-years DMFS by age group**				
**Age group (years)**	**patient N (%)**	**5-years DMFS, N (%)**	**HR (95%CI)**	HR (95% CI)*
≤40	542	352 (64.9)	1.45 (1.19 to 1.77)	1.38 (1.12 to 1.69)
41–50	759	565 (74.4)	1.00 (REF)	1.00 (REF)
51–60	555	394 (71.0)	1.16 (0.94 to 1.43)	1.05 (0.84 to 1.31)
>60	269	180 (66.9)	1.33 (1.03 to 1.70)	1.23 (0.93 to 1.63)
**c. Hazard Ratios and 95% CIs for 5-years BCSS by age group**				
**Age group (years)**	**patient N (%)**	**5-years BCSS N (%)**	**HR (95% CI)**	HR (95% CI)*
≤40	542	460 (84.9)	1.48 (1.09 to 2.01)	1.37 (1.00 to 1.89)
41–50	759	679 (89.5)	1.00 (REF)	1.00 (REF)
51–60	555	479 (86.3)	1.33 (0.97 to 1.82)	1.15 (0.82 to 1.60)
>60	269	231 (85.9)	1.37 (0.93 to 2.01)	1.42 (0.94 to 2.15)

Abbreviations: HR, hazard ratios; DFS, disease-free survival; REF, reference.

Abbreviations: DMFS, distant metastasis-free survival.

Abbreviations: BCSS, breast cancer special-survival.

*Adjusted for tumor stage, histological grade, chemotherapy, endocrine-therapy, trastuzumab and molecular subtype.

### Stratification by molecular subtype

Multivariable Cox proportional hazards regression was used to describe the association between age and 5-year DFS, 5-year DMFS, or 5-year BCSS by molecular subtype as shown in Table 3. After adjusting for tumor stage, histological grade, and treatment, a statistically significant worse 5-year DFS, 5-year DMFS and 5-year BCSS were observed in younger patients with luminal A disease (n = 466) (HR = 2.06 and 95%CI 1.15–3.69, HR = 1.88 and 95%CI 1.04–3.41, HR = 5.85 and 95%CI 1.22–28.01, respectively). Younger women with luminal B (Her2−) showed worse 5-year DFS and 5-year DMFS than the 41 to 50 year old age group (HR = 1.47 and 95%CI 1.05–2.06, HR = 1.51 and 95%CI 1.06–2.15, respectively), whereas no difference in the 5-year BCSS was discovered in patients with this subtype (HR = 1.73, 95%CI 0.87–3.44). For women with the luminal B (Her2+) and HER2 over-expression subtypes, there was no difference in 5-year DFS, 5-year DMFS and 5-year BCSS between the younger and the 41 to 50 year old age groups. For the patients with triple-negative disease, younger age patients showed no significant difference for 5-year DFS, 5-year DMFS, and 5-year BCSS compared to the reference group. Nevertheless, the age >60 group had a statistically significant association with a worse 5-year DFS and 5-year DMFS (HR = 2.16 and 95%CI 1.22–3.82, HR = 1.94 and 95%CI 1.07–3.57, respectively), but not with a 5-year BCSS (HR = 1.94, 95%CI 0.98–3.86), compared with the 41 to 50 year old group.

**Table 3:** Age and 5-year DFS, 5-year DMFS and 5-years BCSS by Molecular Subtype.

Subtype/Age (years)	Patients N(%)	5-year DFS, N(%)	HR (95%CI)*	5-year DRFS, N(%)	HR (95%CI)*	5-year BCSS, N(%)	HR (95%CI)*
**Luminal A**							
≤40	112	85 (75.9)	2.06 (1.15 to 3.69)	87 (77.7)	1.88 (1.04 to 3.41)	104 (92.9)	5.85 (1.22 to 28.0)
41–50	183	161 (69.4)	1.00 (REF)	161 (88.0)	1.00 (REF)	181 (98.9)	1.00 (REF)
51–60	100	81 (81.0)	1.61 (0.86 to 3.03)	82 (82.0)	1.53 (0.81 to 2.91)	96 (96.0)	3.56 (0.65 to 19.5)
>60	71	55 (77.5)	1.94 (0.90 to 4.20)	55 (77.5)	1.96 (0.90 to 4.27)	66 (93.0)	5.02 (0.82 to 30.95)
**Luminal B Her-2** (**−)**							
≤40	186	112 (60.2)	1.47 (1.05 to 2.06)	117 (62.9)	1.51 (1.06 to 2.15)	168 (90.3)	1.73 (0.87 to 3.44)
41–50	232	161 (69.4)	1.00 (REF)	166 (71.6)	1.00 (REF)	216 (93.1)	1.00 (REF)
51–60	168	116 (69.0)	1.12 (0.77 to 1.63)	122 (72.6)	1.09 (0.74 to 1.62)	147 (87.5)	2.24 (1.12 to 4.49)
>60	86	59 (68.6)	0.87 (0.54 to 1.41)	59 (65.1)	0.96 (0.59 to 1.56)	78 (90.7)	1.41 (0.58 to 3.44)
**Luminal B Her-2** (**+)**							
≤40	107	52 (48.6)	1.00 (0.63 to 1.58)	53 (51.0)	1.11 (0.69 to 1.79)	76 (71.0)	0.80 (0.40 to 1.59)
41–50	114	70 (61.4)	1.00 (REF)	75 (65.8)	1.00 (REF)	92 (80.7)	1.0 (REF)
51–60	72	40 (55.6)	1.10 (0.67 to 1.81)	43 (59.7)	1.11 (0.65 to 1.89)	55 (76.4)	1.04 (0.52 to 2.09)
>60	24	14 (58.3)	0.93 (0.44 to 1.99)	15 (62.5)	0.94 (0.42 to 2.08)	19 (79.2)	0.90 (0.31 to 2.59)
**HER-2 over-expression**							
≤40	70	38 (54.3)	1.27 (0.79 to 2.05)	41 (58.6)	1.23 (0.74 to 2.04)	47 (67.1)	1.73 (0.91 to 3.31)
41–50	114	74 (64.9)	1.00 (REF)	77 (67.5)	1.00 (REF)	96 (84.2)	1.00 (REF)
51–60	127	80 (63.0)	0.98 (0.63 to 1.53)	82 (64.6)	0.99 (0.63 to 1.57)	94 (74.0)	1.07 (0.55 to 2.08)
>60	36	21 (58.3)	0.85 (0.42 to 1.70)	21 (58.3)	0.92 (0.45 to 1.88)	31 (86.1)	0.75 (0.24 to 2.29)
**Triple negative**							
≤40	82	52 (63.4)	1.44 (0.86 to 2.40)	54 (65.9)	1.33(0.78 to 2.24)	65 (79.3)	1.13 (0.59 to 2.17)
41–50	116	85 (73.3)	1.00 (REF)	86 (74.1)	1.00 (REF)	94 (81.0)	1.00 (REF)
51–60	88	64 (72.7)	0.87 (0.50 to 1.52)	65 (73.9)	0.90 (0.51 to 1.58)	75 (85.2)	0.73 (0.36 to 1.50)
>60	52	28 (53.8)	2.16 (1.22 to 3.82)	30 (57.7)	1.94 (1.07 to 3.51)	37 (71.2)	1.94(0.98 to 3.86)

*Adjusted for tumor stage, histological grade, chemotherapy, endocrine-therapy, trastuzumab.

## Discussion

As is known, more and more studies have suggested that young age is an independent risk factor for worse disease-free survival and death from more aggressive tumors. In the present study, we used a large cohort of cases obtained from 2,125 women with breast cancer to explore whether young age is a risk factor after controlling for molecular subtypes which have played a pivotal role in predicting prognosis and instructing treatment.

Generally, younger women with breast cancer have been described as those either younger than 35 or younger than 40 years old in different studies. Our study showed that age between 36 and 40 was also a risk factor for a worse 5-year DFS, 5-year DMFS, and BCSS (Table 4), after adjusting for prognostic factors, when dividing the younger age group (≤40) into two groups (≤35 and 36 to 40 years old) and comparing them with the 41 to 50 age group. This finding suggests that an age of 40 years is a reasonable cutoff for defining young age-onset breast cancer, and thus, we defined young patients as those ≤40 years old in the present study. Different from some studies in which younger women have a greater likelihood of having a HER2− type or triple-negative type disease, we found that the luminal B Her(+) and luminal B Her(−) subtypes in our study were the most prevalent subtypes (Table 1)^9, 20, 21^.

**Table 4:** Hazard Ratios and 95% CIs for 5-years DFS, 5-years DMFS and 5-years BCSS by 36–40 age group.

Age group (years)	5-years DFS, N (%)	HR (95%CI)*	5-years DMFS, N (%)	HR (95%CI)*	5-years BCSS, N (%)	HR (95%CI)*
≤35	143 (60.9)	1.39 (1.08 to 1.79)	152 (64.7)	1.34 (1.03 to 1.74)	201 (85.5)	1.23 (0.82 to 1.85)
36–40	196 (63.8)	1.35 (1.06 to 1.71)	200 (65.1)	1.41 (1.10 to 1.80)	259 (84.4)	1.50 (1.04 to 2.17)
41–50	551 (72.6)	1.00 (REF)	565 (74.4)	1.00 (REF)	679 (89.5)	1.00 (REF)

*Adjusted for tumor stage, histological grade, chemotherapy, endocrine-therapy, trastuzumab and molecular subtype.

Just as Cancello *et al*. had reported, younger women show a worse 5-year DFS and 5-year DMFS after adjusting for other prognostic factors compared to older women in our study (Table 2). However, there is borderline risk for 5-year BCSS which is different from some reports that younger women have worse BCSS and OS than older patients^9, 20, 21^. A study published by Emily *et al*. reported similar findings, that young age was not significantly associated with a worse BCSS^23^. However, we consider that the followed-up time may not have been long enough in that study, and thus, 8-year BCSS was obtained in our study and the findings suggest that younger women do have a worse result compared to older women. (data not shown).

Survival seems to be variable between age groups with early-stage breast cancer after stratification by molecular subtypes. Our report indicated that younger women with luminal B HER2(+) and HER2 over-expression types did not have worse 5-year DFS, 5-year DMFS, and 5-year BCSS compared with the 41 to 50 year old age group. Both subtypes have over-expressed or amplified human epidermal growth factor receptor 2, and were advised to undergo targeted treatment with trastuzumab. A similar proportion of these two age groups accepted treatment with chemotherapy (97.1% versus 97.6%) and trastuzumab (2.9% versus 2.4%). Results from a trastuzumab adjuvant trial suggested that young age was not a risk factor for short-term disease-free survival and overall survival for HER2 positive disease; regardless of having had treatment with trastuzumab or not^18^. Similar results had been reported by studies from Italy and California^7, 16^. This finding suggests that young age might seem not to be an independent prognostic factor for these two subtypes.

Triple-negative breast cancer, which is a more aggressive subtype and always considered to be associated with a poor prognosis for younger women, was not an increased risk factor for DFS, DMFS, and BCSS among the younger group compared with the 41 to 50 year old age group after adjusting for other prognostic factor in our study. Similarly, Azim *et al*. had reported a large study with 3,522 patients using gene expression data to investigate the association between age and prognosis of breast cancer by molecular subtype. Their results indicated that there is no significant difference in relapse-free survival between younger and older patients^9^. In addition, Sheridan *et al*. and Kim *et al*. both showed similar recurrence-free survival, OS, and BCSS in younger women compared with older women^19, 22^. A retrospective analysis from China also showed that younger women with triple-negative and Her2 over-expression types of tumors had similar DFS and OS compared with a 40–50 year old group^24^. It can be seen that young age does not increase the risk of recurrence and mortality for patients receiving a curative operation and adjuvant therapy for triple-negative breast cancer.

From the Table 1, we see that despite greater use of adjuvant endocrine therapy, which plays an important role in reducing the risk of recurrence and mortality for younger women compared with the 41 to 50 year old age group (65.3% vs. 62.9%), we still observed a worse outcome for younger patients. This result suggested that endocrine agents in current use are insufficient to overcome age-related differences in the luminal A and luminal B (Her2−) subtypes which are characterized by endocrine-responsive disease. We considered that the inferior outcome of younger women for these two subtypes may result from tamoxifen resistance^25^. Additionally, adherence to treatment is a critical issue for younger patients which may lead to inadequate efficacy and contribute to the inferior outcomes we observed for both subtypes. Hershman and He *et al*. reported that younger women were more likely to discontinue treatment and be non-compliant with their therapy than older women and they associated non-compliance with increased mortality^26–28^. Furthermore, amenorrhea induced by chemotherapy, which has been associated with improved disease-free survival or overall survival among women with premenopausal hormone receptor-positive breast cancer, is less likely to occur in younger women^29, 30^. The combined analysis of the tamoxifen and exemestane trial (TEXT) and the Suppression of Ovarian Function Trial (SOFT) suggested that tamoxifen plus ovarian suppression significantly reduced the risk of recurrence compared with tamoxifen alone for premenopausal women, especially for those <35 years old^31^. However, no benefit was observed from Ovarian Function Suppression (OFS) for luminal A disease in the premenopausal patients, and only luminal B (Her2−) cases receiving chemotherapy had a benefit from exemestane plus OFS^32^. And we are not aware of any similar result reported for young patients with breast cancer. Similarly, Cancello *et al*. found that combination therapy using a luteinizing hormone-releasing hormone (LH-RH) analogue and tamoxifen was significantly correlated with improved DFS when compared with tamoxifen alone for very young patients (age <35 years). However, the benefit of the combination is restricted to luminal B disease but not seen in the women with the luminal A subtype. Further, patients with the luminal B (Her2+) subtype benefits more from the combination when compared to the patients treated with tamoxifen alone^7^. Obviously, younger women seem not to benefit or to benefit less from the suppression of ovarian function if they have the luminal A and luminal B (Her2−) subtypes. In summary, young age plays an important role for the worse outcome in the patients with luminal A and luminal B (Her2−) tumors.

As a heterogeneous disease, it is possible that there are different genotypes that are age-related within molecular subtypes. Recent analysis demonstrated that the results from all three gene expression profile platforms, MammaPrint, genomic grade index, and GENE 76, showed a significant association with disease-free survival in the luminal A and luminal B (Her2−) subgroups, which was independent of age, but not in the Her2 positive disease and the triple-negative subtype. Numerous studies have demonstrated a significantly higher prevalence of BRCA1/2 mutations in younger women with breast cancer, and the BRCA1 mutation has been associated with triple-negative breast cancer for younger women^9^. However, Wang *et al*. reported that there is no significant difference for recurrence-free survival between BRCA1 carriers and non-carriers^33^. More research should be carried out to connect mutations with molecular subtypes and prognosis.

A recent analysis of data from 1,945 patient collected between 2004 and 2007 in British Columbia showed that age <40 years was an independent predictor of recurrence-free survival and overall survival for the luminal subtype but not for triple-negative disease and the Her2−type (HER2 positive)^19^. Similarly, Partridge *et al*. reported a study with 17,575 patients from NCCN data diagnosed prior to 2007, and after clearly defining luminal types, breast cancer special-survival was worse for patients ≤40 years old with luminal A and luminal B subtypes but not those with the triple-negative and HER2 over-expression types^34^. In the present study, we were restricted to molecular sub typing in which Ki67 was included according to the St. Gallen International Expert Consensus to assess whether young age is an independent risk factor for disease-free survival and breast cancer special-survival after stratification. Equally, the findings of our study imply that young age seemed not to increase the risk for survival compared to older age for triple-negative and HER2 over-expression types. Our results support the mounting evidence that the relationship between young age and breast cancer specific-survival varies with molecular subtype. A major strength of our study was the more rigorous definition and explicit classification for the luminal-type that was done in light of the limitations outlined in the most recent studies. After controlling for other prognostic factors for luminal-type, we observed that there were no significant difference in survival with luminal B (Her2+) disease between younger women and older women, although young age seem to be an independent predictor of a worse prognosis for patients with luminal A and luminal B (Her2significantly from the reference).

There are some limitations to our study that should be considered. This study was a single center retrospective analysis, all patients enrolled in the present study were Chinese women and represent an ethnically homogeneous population and results may not apply to other ethnic groups with breast cancer. Although we considered treatments such as chemotherapy and endocrine therapy, we did not have details of the components and regimens of treatments, and failed to collect information about chemotherapy-induced amenorrhea, adherence, suppression of ovarian function, and so on, and the results may be influenced by these residual confounding factors.

In conclusion, our study suggests that the prognostic significance of young age varies with molecular subtype. Younger age was found to be an independent risk factor for survival in breast cancer patients with the luminal A and luminal B (Her2−) subtypes, but not in the luminal B (Her2+), Her2 overexpression, and triple-negative subtypes. The results of our research will contribute to a more accurate assessment of risk for younger women with breast cancer after stratification of disease by molecular subtype and has the potential to improve survival.

## Methods

### Patients

We conducted a retrospective study of women with a primary diagnosis of invasive breast cancer, AJCC stages I, II, and III, (American Joint Committee on Cancer Staging Manual, 7th edition) who had curative surgery in the Fujian Medical University Union Hospital between January 1, 2004 and December 31, 2011. Those who either had had a previous diagnosis of carcinoma or who were missing follow-up information (n = 51) were exclude from the study. For those included, we collected information from their medical records on age at diagnosis, stage at diagnosis (I/II or III), tumor size (≤2 cm or >2 cm), lymph node statue (negative or positive), histologic grade (low/moderate, high, or unknown), estrogen receptor (ER) statue (negative or positive), progesterone receptor (PR) statue (negative or positive), and human epidermal growth factor receptor-2 (HER2) status (negative or positive), Ki67 (low, high, or unknown), received chemotherapy (yes or no), endocrine therapy (yes or no), trastuzumab treatment (yes or no) and molecular subtype (luminal A, luminal B Her2(−), luminal B Her2(+), Her2 over-expression, or triple negative).

### Definitions

Age ≤40 years at the time of breast cancer diagnosis was defined as younger breast cancer patients. The immunohistochemical (IHC) expression of ER, PR, Ki67, and HER2neu were used to classify molecular subtypes, and gene expression profiling was employed if required. An ER and PR expression of more than 1% was considered positive, and expression of more than 20% for PR was classified as high expression. For HER2 status classification, tumors were scored according to the intensity of the cell membrane staining and completeness of cell membrane staining using a 4-tier scale; 0 for no immunoreactivity, 1+ for weak and incomplete membrane staining, 2+ for weak/moderate and complete membrane staining, and 3+ for strong and complete membrane staining. A 3+ tumor was considered HER2 positive, whereas 1+ and 0 tumors were considered HER2 negative. For tumors scored 2+, fluorescent *in situ* hybridization (FISH) results were used, and were considered HER2 positive if FISH was positive, otherwise the HER2 score was negative. The best cutoff point for the Ki67 proliferative index is still under debate. Cheang *et al*. suggested that a level of <14% best correlated with the gene-expression definition of luminal A based on the results in a single reference laboratory. Therefore, we defined expression of 14% or greater as a high Ki67 level and less than 14% as a low level of expression^35^. The histologic tumor grade and biomarker (ER, PR, HER2, and Ki67) status extracted from the pathology reports were used for molecular subtype classification as follow.

Luminal A is ER positive and PR high with HER2 negative and Ki67 low expression or a grade of low/moderate, Luminal B HER2 negative, is ER positive and HER2 negative and either PR low or Ki67 high and grade high), Luminal B HER2 positive, is ER positive and HER2 positive and PR/Ki67 expression is not a factor, HER2 over-expression is ER negative, PR negative, and HER2 positive, and triple-negative is ER negative, PR negative, and HER2 negative^36, 37^.

### Analyses

We stratified by age at diagnosis (≤40, 41 to 50, 51 to 60, and >60 years old), and the 41 to 50 age group acted as the reference group; it had the highest number of patients and allowed us to compare the difference between young women and older women. Analyses were performed with SAS software (version 9.4, Institute, Cary, NC), and all two-sided P-values less than 0.05 were considered statistically significant. We used Chi-square tests to compare the clinical and pathological characteristics between the reference group and other age groups. The follow-up duration was calculated from the date of diagnosis until the date of death or at the end of the study period (January 1, 2016). We report 5-year disease-free survival (DFS), 5-year distant metastasis-free survival (DMFS), and 5-year breast cancer-specific survival (BCSS). The DFS was defined as the time from diagnose to the detection of loco-regional recurrence, distant metastasis, or death due to any cause. We calculated DMFS from the time of diagnose to the appearance of any distant metastases, including contralateral axillary and supraclavicular lymph nodes. The BCSS was defined as the time from diagnose to death from breast cancer. Multivariable Cox proportional hazards regression was developed to estimate the hazard ratios (HR) and 95%CI for the relationship between the age group and 5-year DFS, 5-year DMFS or 5-year BCSS. Adjustments were then carried out on multivariate analyses which included diagnosis (stage I/II or III), histological grade (low/moderate or high), chemotherapy (yes or no), endocrine-therapy (yes or no), trastuzumab treatment (yes or no), and molecular subtype (luminal A, luminal B (Her2+), luminal B (Her2−), HER2 over-expression, and triple-negative). Above all, we conducted separate analyses within each molecular subtype, and adjusted for other prognostic factors, of which stage at diagnosis (I/II or III), tumor grade (low/moderate or high), chemotherapy (yes or no), endocrine therapy (yes or no), or trastuzumab treatment (yes or no) were included.

### Data Availability

The datasets generated during and/or analyzed during the current study are available from the corresponding author on reasonable request.

### Ethical standards

All methods were carried out in accordance with relevant guidelines and regulations. Our study were approved by Fujian Medical University Union Hospital Ethics committee.
